# Extraction of Cathepsin D-Like Protease from Neon Flying Squid (*Ommastrephes bartramii*) Viscera and Application in Antioxidant Hydrolysate Production

**DOI:** 10.3390/biom9060228

**Published:** 2019-06-12

**Authors:** Kaiqiang Zhang, Rongbian Wei, Ru Song

**Affiliations:** 1 School of Food Science and Pharmacy, Zhejiang Ocean University, Zhoushan 316000, China; zhangkaiqiang3@163.com; 2 School of Marine Science and Technology, Zhejiang Ocean University, Zhoushan 316022, China; rbwei@zjou.edu.cn

**Keywords:** neon flying squid viscera, isoelectric solubilization/precipitation, ultra-membrane filtration, cathepsin D-like protease, property, half-fin anchovy (*Setipinna taty*) hydrolysates, antioxidant

## Abstract

A protease from neon flying squid (*Ommastrephes bartramii*) viscera (SVCE3(f)) was partially purified by isoelectric solubilization/precipitation combined with ultra-membrane filtration (ISP-UMF). Two protein bands of 45 and 27 KDa were determined by SDS-PAGE assay. The protease characteristic of the protein band of 45 KDa was confirmed using casein zymography analysis. The result of UPLC-ESI-MS/MS suggested that the band of 45 KDa could be a cathepsin D-like protease. This cathepsin D-like protease showed an optimum pH of 3.0 and optimum temperature of 60 °C when casein was used as s substrate. Furthermore, its protease activity was stable at 30–50 °C and under a pH range of 1.0–5.0, maintaining about 60% of its initial activity. SVCE3(f) can digest half-fin anchovy (*Setipinna taty*) to generate antioxidant hydrolysates (HAHp-SEs). The degree of hydrolysis (DH) of HAHp-SEs increased along with the hydrolysis time and reached stability after 60 min of digestion. HAHp-SEs(30) with relatively lower DH exhibited the highest DPPH radical scavenging activity as compared with other HAHp-SEs. However, a stronger hydroxyl radical scavenging activity and greater reducing power were observed for HAHp-SEs that underwent higher DH. Accordingly, the partially purified cathepsin D-like protease of neon flying squid viscera using ISP-UMF could have potential application in antioxidant hydrolysates production.

## 1. Introduction

The total amount of squid captured by the Chinese squid fishing fleets annually is more than 300,000 tons in recent years, accounting for around one-sixth of the global oceanic squid production [1]. Neon flying squid (*Ommastrephes bartramii*), widely distributed in the North Pacific, is one of the most captured squid species for Chinese squid jigging fishery [2]. Like many other squid species, neon flying squid is mainly commercialized as fresh or frozen mantle, thus, plenty of squid viscera, which contributes approximately 30% of the total weight of the squid, may cause environmental problems if discarded. These materials, however, are rich sources of various enzymes that would have diverse applications in a wide range of industries such as food, pharmaceutical, and leather industries [3]. To date, many enzymes such as cysteine proteinase, trypsin, cathepsin C, cathepsin D, lipase, and aminopeptidase have been identified from the by-products of squid species like *Dosidicus gigas* or *Todarodes pacificus* [4,5,6,7,8,9]. Enzymes, such as chromotrypsin and trypsin, were also characterized from cuttlefish, which belong to the same family as neon flying squids [3,10,11].

Usually, the conventional separation techniques applied to obtain enzymes from organisms include salt and organic precipitation, chromatography, two-phase, or three-phase separations [12]. Properly employing these methods alone, or a combination of them, may achieve separation of proteases from plants or animals effectively. However, most of these conventional procedures of enzyme purification are either time-consuming, involve expensive reagents with a potential residue of organic solvents, or are difficult to scale-up [12,13]. Isoelectric solubilization/precipitation (ISP) is a pH-shift process that induces water solubility of a protein based on its isoelectric behavior when subjected to pH changes [14]. When the pH is close to the isoelectric point (pI) of proteins, the protein–protein hydrophobic attraction is greater than the protein–water electrostatic attraction, resulting in isoelectric precipitation. On the other hand, when the pH is changed away from the pI of a protein, the protein–water attraction is stronger than protein–protein electrostatic repulsion resulting in isoelectric solubilization [15]. The ISP process has been used as a convenient approach to recover high-quality protein isolates from food by-products or under-utilized sources, such as PSE (pale, soft, exudative)-like chicken [16], krill [17], and Atlantic croaker muscle [18]. However, the reports are limited for proteases recovered from fish viscera by ISP.

Proteases, one of the three largest groups of industrial enzymes [13], have great contributions to produce bioactive ingredients in the enzyme industry. Fish viscera are a rich source of digestive enzymes, especially proteases, and the extracted proteases can be used as processing aids in food industries [19]. For example, the use of acidic crude protease from the viscera of zebra blenny (*Salaria basilisca*) was applied in the gelatin production process [20]. A source of alkaline proteases extracted from the viscera of *Colossoma macropomum* was used as laundry detergent [21]. Crude alkaline proteases from the viscera of *Portunus segnis* were regarded as the alternative deproteinization step to chemical treatment to extract crab and shrimp chitin [22]. Zebra protein hydrolysates obtained by treatment with crude alkaline protease extracts from zebra blenny, sardinella, and smooth hound viscera showed good in vitro antioxidant activities [23].

In our previous study, we reported the antioxidant activity of half-fin anchovy (*Setipinna taty*) hydrolysates after digestion by commercial pepsin [24]. However, the potential utilization of extracted proteases from squid viscera to generate antioxidant half-fin anchovy hydrolysates was not yet investigated. The aim of the present study was to (1) develop an ISP combined with ultra-membrane filtration process (ISP-UMF) to isolate protease from neon flying squid viscera and (2) to apply the extracted protease to generate antioxidant half-fin anchovy hydrolysates. Hence to provide a potential method to solve the environmental problems caused by discarded squid viscera and for the further utilization of the extracted protease, in a long run, for the valorization of the by-products in the squid processing industry.

## 2. Materials and Methods

### 2.1. Materials

Fresh neon flying squid (*O. bartramii*) viscera were supplied by Zhejiang Fudan Tourism Food Co., Ltd. (Zhoushan, China). The viscera were packaged in polyethylene bags and transported to the laboratory at 4 °C with ice bags. Upon arrival, the viscera were rinsed thoroughly with cold distilled water, and then immediately used for crude enzymes extraction. Sodium dodecyl sulfate-polyacrylamide gel electrophoresis (SDS-PAGE) marker was purchased from Beyotime Biotechnology (Shanghai, China). Bovine serum albumin (BSA) was provided by Shanghai EKEAR BioTech Co., Ltd. (Shanghai, China). Other reagents were obtained from Sinopharm Chemical Reagent Co., Ltd. (Shanghai, China).

### 2.2. Extraction of Crude Enzymes

**Figure 1:**
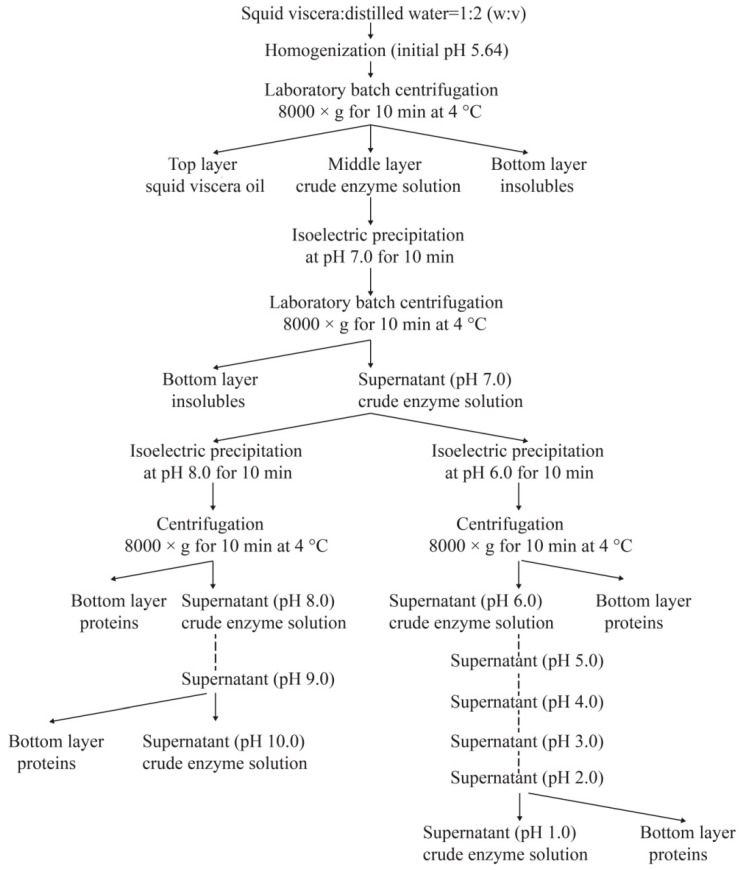
Schematic illustration of the procedures for extraction of proteases from neon flying squid viscera using isoelectric solubilization/precipitation.

After the removal of ink sac, squid skin, and other irrelevant components, the viscera were cut into small pieces (the length was about four centimeters) by scissors. Two hundred grams of viscera were mixed with cold distilled water at a ratio of 1:2 (*w*/*v*), and then homogenized at a speed of 10,000 rpm for an interval of 1 min with five intermissions (30 s each time) by a homogenizer (TM-767, Zhongshan, China). After keeping at 4 °C for 10 min, the homogenates were centrifuged at 8000× *g* for 10 min, the supernatants were then collected and pretreated according to the procedures described in Figure 1, to prepare squid viscera crude enzymes (SVCEs) through the ISP process.

### 2.3. Protease Activity and Specific Activity Assays

Protease activity of supernatants with different pH values was determined by the method described by Uddin, Ahn, Kishimura, and Chun [13] using casein as the substrate with minor modifications. In brief, the casein solutions (10 mg/mL) with pH 3.0 (dissolved in 0.05 M lactic acid buffer), pH 7.5 (dissolved in 0.05 M phosphate buffer), and pH 10.5 (dissolved in 0.05 M boric acid buffer) were used as substrates for acid protease, neutral protease, and alkali protease activities assay, respectively. An aliquot of 1.0 mL of supernatant (obtained according to Figure 1) was mixed with the corresponding casein substrate and incubated at 40 °C for 10 min. The reaction was terminated by the addition of 2.0 mL of 0.4 M trichloroacetic acid and settled for 10 min at room temperature. After centrifugation at 5000 rpm for 10 min, the supernatant (1.0 mL) was mixed with 5 mL of 0.4 M Na_2_CO_3_ and 1 mL of Folin–Ciocalteu reagent. After an incubation of 20 min at 40 °C, the absorbance was determined under 680 nm in a spectrometer (721G, Analytical Instrumental, Shanghai, China). One unit of protease activity was defined as the amount of enzyme required for the liberation of 1 μmol tyrosine per min from casein.

The specific activity of protease was expressed as protease activity per protein (mg) (U/mg protein). Protein concentration was determined by a Bradford assay [25] using bovine serum albumin as a standard.

### 2.4. Purification of Crude Enzymes

The obtained fraction by ISP with higher activity of protease and specific activity were further separated using a membrane system (WTM-1812G, Hangzhou Watech Membrane Engineering Co., Ltd., Hangzhou, China) equipped with an 8 KDa filter membrane (GM1812C-34D, GE Water and Process Technologies (Wuxi) Co. Ltd., China). The fraction with molecular weight (MW) higher than 8 KDa was collected (named as SVCE3(f)) and used for SDS-PAGE and casein zymography assays to determine the purification and protease activity.

SDS-PAGE was performed according to the method of Laemmli [26]. Briefly, the electrophoresis was carried out at an inconstant voltage system of 80 V for 30 min on the pH 6.8 stacking gel (5% *w*/*v,* 83.0 mm × 15.0 mm × 1.0 mm), and then 120 V for 2 h on the pH 8.8 separating gel (12% *w*/*v*, 83.0 mm × 65.0 mm × 1.0 mm). The gel was stained for 4 h with 0.05% Coomassie Brilliant Blue R-250 in 45% methanol–10% acetic acid and then de-stained for 2 h with 5% methanol–7.5% acetic acid. A low molecular weight calibration kit, with 116.0, 66.2, 45.0, 35.0, 25.0, 18.4, and 14.4 KDa used as the marker, was performed at the same conditions. A linear regression of markers was obtained as *y* = −1.4123*x* + 2.1718 (R^2^ = 0.9800), according to the lg (molecular weight of marker) (*y*) and relative migration (cm) (*x*). The molecular weight of separated bands of SVCEs was calculated according to the linear regression of markers.

Zymography analysis in order to identify protease from separated bands was conducted by the method of Khaled et al. (2011) [27] with minor modifications. In brief, after electrophoresis of SVCE3(f) on SDS-PAGE, the gel was submerged in 1% (g/mL) casein solution (dissolved with 100 mM pH 3.0 lactic acid buffers) and incubated at 37 °C for 12 h. After washing, the gel was stained for 3 h with 0.05% Coomassie Brilliant Blue R-250 in 45% methanol–10% acetic acid and then de-stained for 30 min with 5% methanol–7.5% acetic acid. The presence of protease activity was indicated by the development of a clear band on the blue background of the gel.

### 2.5. Identification of Enzyme by UPLC-ESI-MS/MS

The separated bands of SVCE3(f) in SDS-PAGE were cut off and hydrolyzed with trypsin at 37 °C for 15 h. The hydrolysates of SVCE3(f) were isolated on a C_18_ column (3 µm, 250 mm × 75 µm, 120 Å, Eksigent) using a UPLC system (Waters Corporation, Milford, MA, USA) coupled with electrospray ionization Fourier transform ion cyclotron resonance mass spectrometry (ESI-FTICR) by the instrument of Thermo Fisher Scientific, Bremen, Germany. Solvent A, 0.1% (*v*/*v*) of formic acid in distilled water, and solvent B, formic acid 0.1% (*v*/*v*) in acetonitrile were used for elution. Gradient elution was carried out according to the following process: 0–3 min, isocratic gradient 5% B; 4–60 min, linear gradient 5–40% B; 61–66 min, isocratic gradient 85% B; 67–90 min, isocratic gradient 5% B at a flow rate of 3 µL/min. The separated fragments were then analyzed in a positive-ion mode by electrospray ionization tandem mass spectrometry (ESI-MS) (Q Exactive, Thermo Fisher Scientific, USA). Spectra were recorded over the mass-to-charge ratio (*m*/*z*) range of 100–1800. The data collected were analyzed with Mascot Software under the search type of MS/MS ion search and compared with protein sequences in the protein database of Teuthida and Coleoidea in NCBI (www.ncbi.nlm.nih.gov, Bethesda, MD, USA), which contain 5045 and 48,638 protein sequences, respectively.

### 2.6. Protease Features and Stability of SVCE3(f)

#### 2.6.1. pH Range and Stability

The optimum action pH range of SVCE3(f), semi-purified protease fraction, was determined using casein as a substrate by assaying the proteolytic activity in different pH (1.0–10.0) after incubation at 40 °C for 10 min. The buffer of 50 mM lactic acid buffer (1.0–4.0), 50 mM phosphate buffer (5.0–8.0) and 50 mM boric acid buffer (9.0–10.0) were used, respectively. The protease activities were measured immediately by the standard methods as mentioned above, after treatment with substrate. The effect of pH on protease stability was evaluated by incubating SVCE3(f) at various pH values (1.0–10.0) using different buffers as mentioned above for 60 min at 40 °C. The relative activity was determined based on the residual protease activity of SVCE3(f) after incubation.

#### 2.6.2. Temperature Tolerance Profile and Stability

After incubation at different temperatures (30–70 °C) for 10 min in lactic acid buffer (50 mM, pH 3.0), the protease activity of SVCE3(f) was measured according to the method described in Section 2.3. The stability of SVCE3(f), incubated at different temperatures (30–70 °C) for 60 min, was evaluated based on its residual protease activities under standard assay conditions.

### 2.7. Preparation of Antioxidant Half-Fin Anchovy Hydrolysates using SVCE3(f)

In our previous study, we prepared antioxidant half-fin anchovy hydrolysates with pepsin [24]. In this study, half-fin anchovy was digested by SVCE3(f) instead of pepsin. The minced half-fin anchovy meat was mixed with four-fold volume of deionized water (*w*/*v*), followed by adjusting the pH to 3.0 (optimum pH for SVCE3(f)) with 6 M HCl. Then, SVCE3(f) was added to the mixture at a ratio of 1% (weight of dry SVCE3(f))/(weight of wet meat), and incubated at 60 ± 2 °C (optimum temperature for SVCE3(f)) for 10, 30, 60, and 90 min, respectively. Finally, the reaction solutions were heated at 95 °C for 10 min to inactivate protease, followed by cooling down to room temperature. The mixtures were centrifuged at 8000× *g* for 10 min (TGL-16C, Shanghai, China). The supernatants derived from different incubation times were collected and designated as HAHp-SEs(10), HAHp-SEs(30), HAHp-SEs(60), and HAHp-SEs(90), respectively.

### 2.8. DH Assay

The degree of hydrolysis (DH) was defined as the percent ratio of the α-amino acid content in the total nitrogen content [28]. The α-amino acid content of HAHp-SEs(10), HAHp-SEs(30), HAHp-SEs(60), and HAHp-SEs(90 was determined by a formal titration method [29] with few modifications. Briefly, 5 mL of sample was diluted with distilled water into 100 mL. Subsequently, 20 mL of the dilution was blended with 60 mL of distilled water. The mixture was titrated to pH 8.20 using 0.05 M NaOH, followed by addition of 10 mL of formaldehyde (20%, *v*/*v*). Then, the mixture was titrated to pH 9.20 with 0.05 M NaOH. Distilled water, instead of hydrolysates, diluted in the same reaction conditions was used as the blank group. The α-amino acid content was calculated as in Equation (1).

α-amino acid content (g/mL) = (V_1_ − V_0_) × C_NaOH_ × 0.014 (1)

where V_1_ was the volume of 0.05 M NaOH consumed for sample, titrating from pH 8.20 to pH 9.20; V_0_ was the volume of 0.1 M NaOH consumed in the blank group; C_NaOH_ was the concentration (mol/L) of the aqueous NaOH used; and 0.014 represents the mass (g) of nitrogen which is equivalent to 1 mL of 1 mol/L NaOH.

Total nitrogen in hydrolysates was determined by the Kjeldahl method [30], and the protein content was calculated by a conversion coefficient of 6.25 of total nitrogen. The DH was measured according to Equation (2).

DH (%) = (α-amino acid content/Protein content) × 100(2)

### 2.9. In Vitro Antioxidant Activity of HAHp-SEs

#### 2.9.1. DPPH Radical Scavenging Activity

The capacities of HAHp-SEs(10), HAHp-SEs(30), HAHp-SEs(60), and HAHp-SEs(90) for scavenging DPPH radicals at the same concentration of 1 mg/mL were evaluated by the method described by Song, Wei, Zhang, Yang, and Wang [31] with minor modifications. Briefly, 300 μL of 99.5% ethanol was mixed with 600 μL of samples. After the addition of 30 μL 0.02% DPPH (dissolved in 99.5% ethanol), the mixtures were blended vigorously and kept at room temperature in the darkness for 60 min. The absorbance of the resulting solution (A_s_) was measured at 517 nm with a visible light spectrophotometer (721G, Analytical Instrumental, Shanghai, China) using a micro-cuvette. The DPPH radical scavenging activity was determined by the following equation.

DPPH radical scavenging activity (%) = [A_c_ − (A_s_ − A_b_)]/A_c_ × 100 (3)

where A_c_—replaced sample with equal volume of distilled water; A_b_—replaced 30 μL 0.02% DPPH with 30 μL of 99.5% ethanol.

#### 2.9.2. Reducing Power

The reducing power abilities were measured for HAHp-SEs(10), HAHp-SEs(30), HAHp-SEs(60), and HAHp-SEs(90) at the concentration of 1 mg/mL by the method described in our previous study [24] with slight modifications. Briefly, 200 μL of samples were blended with 100 μL of 0.2 M sodium phosphate buffer (pH 6.6) and 100 μL of 1% potassium ferricyanide in a plastic centrifugal tube. The mixture was incubated at 50 °C for 20 min, followed by addition of 100 μL of 10% (*w*/*v*) trichloroacetic acid. Subsequently, 100 μL of 0.1% (*w*/*v*) ferric chloride was added to the mixture and incubated at room temperature for 10 min. The absorbance of the resulting solution was detected at 700 nm using a visible spectrophotometer (721G, Analytical Instrumental, Shanghai, China) with a micro-cuvette. A higher absorbance suggested a better reducing power.

#### 2.9.3. Hydroxyl Radical Scavenging Activity

The hydroxyl radical scavenging activity of HAHp-SEs(10), HAHp-SEs(30), HAHp-SEs(60), and HAHp-SEs(90) were evaluated at the same concentration of 1 mg/mL with the method of de Avellar et al. [32] with a few modifications. Briefly, 70 μL of samples, 70 μL of 0.75 mM 1, 10-phenanthroline, and 70 μL of 0.75 mM FeSO_4_ were added into a 1.5 mL plastic centrifugal tube. After the addition of 140 μL of 0.2 M sodium phosphate buffer (pH 7.4) and 70 μL of 0.12% (*v*/*v*) H_2_O_2_, the mixtures were incubated at 37 °C for 60 min. Finally, the absorbance of a sample of the resulting solution (A_s_) was measured at 536 nm using a visible spectrophotometer (721G, Analytical Instrumental, Shanghai, China) with a micro-cuvette. The hydroxyl radical scavenging activity was expressed as:

Hydroxyl radical scavenging activity (%) = (A_s_ − A_p_)/A_b_ × 100 (4)

where A_p_—replaced sample with equivalent volume of distilled water; A_b_—replaced sample and H_2_O_2_ with 140 μL of distilled water.

### 2.10. Statistical Analysis

Data in this experiment were expressed as mean ± standard deviation (*n* = 3). One-way analysis of variance (ANOVA) followed by Duncan’s multiple-range test was used to evaluate the significant differences between samples (*p* < 0.05) using SPSS software (SPSS Statistical Software 19.0, Inc, Chicago, IL, USA).

## 3. Results and Discussion

### 3.1. Protease Activity and Specific Activity of SVCEs

**Figure 2:**
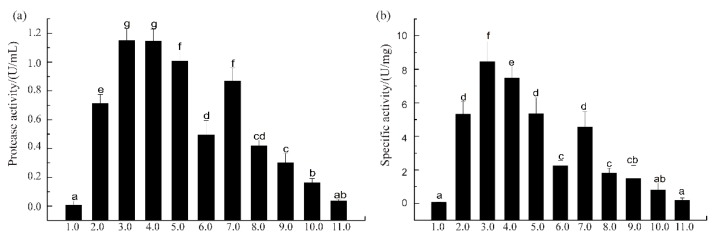
Protease activity (**a**) and specific activity (**b**) of the extracted crude enzymes by isoelectric solubilization/precipitation (ISP) method, using casein as substrate. Different letters indicate significant differences between two compared groups (*p* < 0.05).

Proteins are reported to exhibit maximal solubility between pH 7 and 10, while exhibiting a low solubility below pH 4 [33]. The principle of ISP is to solubilize proteins at high or low pH, removing debris and precipitating the proteins near their isoelectric points [33]. Squid viscera have wide biotechnological potential as a source of digestive enzymes [3,10,13,34]. The suspensions of SVCEs, extracted by duplicated stepwise ISP procedure according to the scheme of Figure 1, demonstrated different protease activities and specific activities, as shown in Figure 2.

When SVCEs were extracted under extreme acidic or alkaline pH conditions, such as pH 1.0 and pH 10.0, the protease activity of SVCEs, as well as their specific activity, was much lower compared to other groups of pH ranges. The largest protease activities of SVCEs were detected for the group with extraction pH ranges of 3.0–4.0 (Figure 2a). Furthermore, the SVCEs obtained at pH 3.0, designated as SVCE3, showed the highest specific activity, i.e., 8.21 U/mg (Figure 2b).

In this study, during the process of squid viscera suspension production, when the pH shifted to 3.0, some proteins or enzymes, such as myofibrillar proteins with isoelectric point of 5.3 [35], were precipitated. This could contribute to the recovered supernatant having higher amounts of potential proteases. With the purpose of cutting off smaller soluble molecules, the supernatant of SVCE3 was filtered by an 8 KDa ultra-membrane filtration system, and the collected filtrate (>8 KDa) was designated as SVCE3(f). The protease activity and specific activity of extracted enzymes at different steps are summarized in Table 1.

SVCE3 demonstrated increase of enzymatic activities by 2.35- and 3.68-fold for protease activity and specific activity, respectively, compared to SVCE (extracted at initial pH 5.64). After membrane filtration (8 KDa), the specific activity of SVCE3(f) was further enhanced to 8.92 ± 0.81 U/mg with 54.55 ± 9.09% protein recovery. Therefore, it can be seen that the approach of ISP-UMF should be a convenient and effective way to extract proteases from squid viscera.

### 3.2. SDS-PAGE and Zymography Analysis

**Figure 3:**
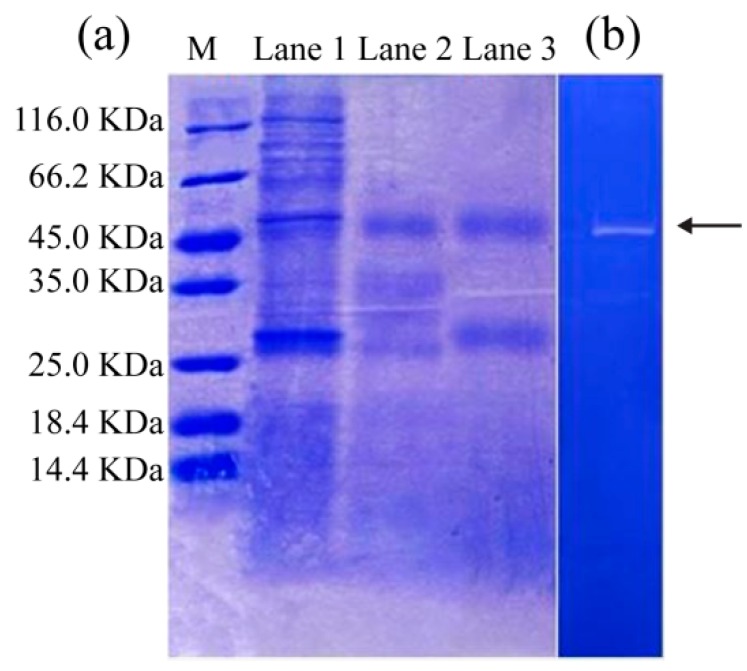
SDS-PAGE of (**a**) SVCE and isolated extracts derived from ISP at pH 3.0 and filtration by 8 KDa ultra-membrane. M: molecular standard marker; Lane 1-SVCE, extracted at initial pH 5.64; Lane 2-SVCE3, obtained at pH 3.0 using ISP; Lane 3- SVCE3(f), produced by filtration of SVCE3 through a membrane with 8 KDa cutoff. (**b**) Casein zymography of SVCE3(f).

SDS-PAGE analysis was carried out to evaluate the molecular weight profiles of SVCEs and their derived fractions (Figure 3).

SVCE, which was extracted at an initial pH of 5.64, contained several protein bands, with molecular weights from 25.0 to 116 KDa (Lane 1 in Figure 3a). After ISP at pH 3.0, a number of protein bands with relatively large MWs disappeared, and only two protein bands with MWs of about 45 and 27 KDa were clearly presented in SVCE3 (Lane 2 in Figure 3a). Similar results were found for SVCE3(f) (Lane 3 in Figure 3a).

The zymography profile can be used to identify the protease property of the protein band. A clear zone on the dark background is identified as the activity of a protease against its substrate (casein) on the polyacrylamide gel [12]. It is clear in Figure 3b that the protein band of SVCE3(f) with MW of 45 KDa (designated as SVCE3(f)-1) appeared on the zymography gel, suggesting its protease characteristics of hydrolysis to casein. By comparison, no zone was observed for the protein band with MW of 27 KDa in Figure 3b. The result of Figure 3b suggested that at least one kind of protease existed in SVCE3(f). Eventually, the band of interest, SVCE3(f)-1, was cut off and used for further MS/MS analysis.

### 3.3. Identification of Protease in SVCE3(f) by UPLC-ESI-MS/MS

A technique of in-gel digestion with trypsin was used to obtain peptides and then identified using mass spectrometry [34]. Possible peptide sequences derived from the trypsin digestion of SVCE3(f) were analyzed with UPLC-ESI-MS/MS. The peptide fragment sequences detected by MS/MS were searched against the Teuthida and Coleoidea protein databases in NCBI (Table 2).

As seen in Table 2, several peptide fragments derived from SVCE3(f) trypsin digest partially matched to 5 and 12 proteins in Teuthida and Coleoidea protein databases, respectively. Furthermore, SVCE3(f) showed the greatest matching degree with cathepsin D (from *T. pacificus*) than other proteins in both Teuthida and Coleoidea protein databases, which could be inferred by the highest protein score and coverage ratio. The result of Table 2 indicated that SVCE3(f) could be a cathepsin D-like protease.

The appearance of a protein band in SDS-PAGE will depend on the conditions of the gel electrophoresis [36], however, the result of SDS-PAGE could contribute to reference molecular weight range of interesting proteins. In this study, the cathepsin D-like protease of SVCE3(f) showed similar molecular weights with several identified and characterized cathepsin D homologs, such as a 45 KDa cathepsin D purified from the deuterostome *Asterias rubens* [37], an ovary cathepsin D with 43 KDa from *Xenopus laevis* [38], and a 43 KDa cathepsin D from the mussel *Lamellidens corrianus* [39]. By comparison of molecular mass, we also found that this cathepsin D-like protease existing in SVCE3(f) had different molecular weights compared to some other cathepsin D obtained from squid. Komai et al. (2004) [7] reported a cathepsin D from the hepatopancreas of Japanese common squid (*T. pacificus*) with MW 36.5 KDa. A heterodimer of cathepsin D with molecular masses of approximately 10 and 28 KDa was identified from the digestive gland of the pelagic squid *Todarodes sagittatus* [40]. These documents clearly indicated the mass diversity of cathepsin D protease obtained from different squid species and different tissues.

### 3.4. Protease Features and Stability of SVCE3(f)

Cathepsin D is an endoproteolytic aspartic proteinase, which has been found to have various degradation functions within cells [41]. For example, a cathepsin D from herring muscle (*Clupea harengus*) had optimal activity at pH 2.5 with hemoglobin as the substrate and digested the β-chain of oxidized insulin in the preferential cleavage sites at Leu15–Tyr16, (47%), Tyr16–Leu17 (34%), and Ala14–Leu15 (18%) [42]. However, the activities of cathepsin D or cathepsin D-like proteases were inhibited by pepstatin and chymostatin [42,43]. Besides intracellular protein hydrolysis [44,45], cathepsin D is involved in digestion of food proteins in invertebrates [45,46]. It is possible to recover large amounts of cathepsin D-like protease from neon flying squid viscera through ISP-UMF and to utilize it in exogenous protein digestion. In the present study, the pH and temperature properties of SVCE3(f), semi-purified cathepsin D-like protease from ISP-UMF, were therefore evaluated, aiming at suggesting suitable enzymatic conditions for hydrolysate production.

#### 3.4.1. pH Profile and Stability

**Figure 4:**
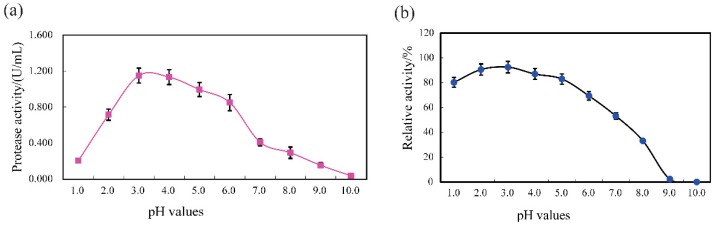
pH profile (**a**) and pH stability (**b**) of SVCE3(f) from squid viscera measured with casein substrate at 40 °C.

SVCE3(f) displayed activity over a broad pH range of 3.0–5.0 with an optimum at pH 3.0 with casein substrate (Figure 4a). The protease activity of SVCE3(f) sharply declined when the pH increased beyond 6.0. Acidic proteases from aquatic sources usually demonstrated high activity between pH 2.0 and 4.0, while alkaline digestive proteases were most active between pH 8.0 and 10.0 [47]. The acidic property of SVCE3(f) was consistent with cathepsin D homologs, which have been known to function at acidic pH [48,49]. Similarly, a new cathepsin D from Japanese common squid viscera demonstrated optimal activity at pH 3.5, pH 2.2, and pH 3.0 for the substrates of acid-denatured hemoglobin, acid-denatured casein, and MOCAc-GKPILFFRLK(Dnp)-d-R-NH2, respectively [7]. Rojo, Sotelo-Mundo, García-Carreño, and Gráf [36] found one kind of cathepsin D from American lobster displayed the highest proteolytic activity at pH 3.0. A similar result was also observed for cathepsin D from the mussel *L. corrianus* with optimum pH 3.5 [39]. The pH stability analysis, shown in Figure 4b, suggested that SVCE3(f) was highly stable at pH ranges of 1.0 to 5.0, maintaining more than 80% of its original protease activity after incubation at different pHs for 60 min. Therefore, SVCE3(f) could be used as a good source of acidic protease in certain industrial applications with requirements for high acid conditions.

#### 3.4.2. Temperature Tolerance and Stability

**Figure 5:**
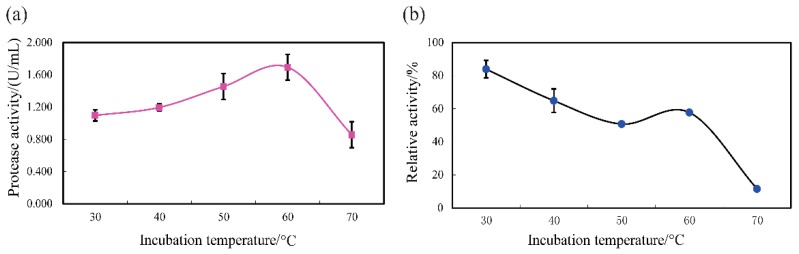
Thermal profile (**a**) and thermal stability (**b**) of SVCE3(f) from squid viscera. Thermal profile was measured with casein as substrate at pH 3.0.

Optimum temperature is an operational parameter for protease activity, depending on the conditions of the assay [50]. In this study, the effects of temperature on the protease and specific activities of SVCE3(f) were determined after heated at different temperatures for 10 min at pH 3.0 with casein substrate, as shown in Figure 5. 

The protease activity was enhanced for SVCE3(f) with increased temperatures, and the optimum temperature was 60 °C for SVCE3(f) (1.69 U/mL) (Figure 5a). However, a dramatically decreased activity was observed above 60 °C. This may be due to thermal denaturation [20]. A similar result was stated for digestive enzymes from squid viscera, which showed the best protease activity at 60 °C with casein solution substrate [13].

The thermal stability of SVCE3(f) is shown in Figure 5b. After incubation at different temperatures for 60 min at pH 3.0, it can be seen that the relative protease activity of SVCE3(f) remained above 50% with treating temperatures ranging from 30 up to 60 °C, while relative enzymatic activity was almost lost after incubation at 70 °C. SVCE3(f) showed stronger thermal stability than other fish-viscera-derived proteases, such as an aspartic protease (17 KDa) from the defatted viscera of sardinella (*Sardinella aurita*) with more than 50% of enzymatic activity after heating for 30 min at 50 °C but unstable above 50 °C [27], an aspartic proteinase obtained from the hepatopancreas (liver) of Japanese common squid (*T. pacificus*) decreased its activity rapidly at 50 °C [7], and a cathepsin D-like enzyme (40 KDa) from Atlantic cod (*Gadus morhua L.*) liver was almost completely inactivated after 15 min at 40 °C [51].

### 3.5. Hydrolysis of Half-Fin Anchovy with SVCE3(f)

**Figure 6:**
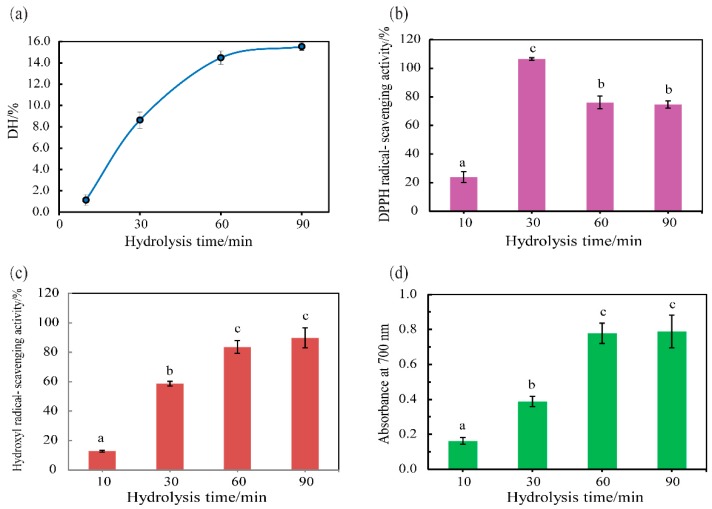
Antioxidant activity of half-fin anchovy hydrolysates digested with SVCE3(f) at different times. (**a**) Degree of hydrolysis, (**b**) DPPH scavenging activity, (**c**) hydroxyl radical scavenging activity, and (**d**) ferric reducing power. Data were presented as mean ± SD (*n* = 3). Different letters represent significant differences (*p* < 0.05).

SVCE3(f) was used to hydrolyze half-fin anchovy to produce antioxidant hydrolysates. The results were shown in Figure 6.

During protein hydrolysis, the DH has a strong effect on the functional property of hydrolysates, and therefore would determine the potential application of protein hydrolysates [52]. The DHs of resulting half-fin anchovy hydrolysates digested by SVCE3(f) under pH 3.0 and incubation temperature of 60 °C for 10, 30, 60, and 90 min, namely HAHp-SEs(10), HAHp-SEs(30), HAHp-SEs(60), and HAHp-SEs(90), were 1.29%, 8.63%, 14.47%, and 15.52%, respectively (Figure 6a). Clearly, the DH was increased along with the increase of incubation time and then reached a relatively stable stage. As shown in Figure 6b–d, in vitro antioxidant activities of the four hydrolysates were compared by the ability of scavenging DPPH and hydroxyl radicals, and ferric ion reducing ability. With the hydrolysis time of 10 min, the DPPH and hydroxyl radical scavenging activities of HAHp-SEs(10) were 23.88 ± 3.79% and 12.76 ± 0.62%, respectively. Similarly, the lowest reducing power was 0.16 ± 0.02 for HAHp-SEs(10). The weak in vitro antioxidant activity of HAHp-SEs(10) may be due to its low DH, which likely indicates the lack of peptide fragments or amino acids generated.

HAHp-SEs(60) and HAHp-SEs(90) demonstrated similar in vitro antioxidant activities on scavenging DPPH and hydroxyl radicals and ferric ion reducing ability (*p* > 0.05). This was in accordance with their DHs (Figure 6a). Furthermore, HAHp-SEs showed increased hydroxyl radical scavenging activity and reducing power ability in pace with increased incubation time (from 10 to 60 min). By comparison, the highest DPPH scavenging activity of HAHp-SEs was found for HAHp-SEs(30) (*p* < 0.05), and the DPPH radical scavenging activity decreased to 76.10 ± 4.45% for HAHp-SEs(60) and 74.59 ± 2.48% for HAHp-SEs(90), respectively. The result of Figure 6 indicated that the increases of DH could contribute to improving the antioxidant activities of scavenging hydroxyl radical and reducing power; whereas further hydrolysis could decrease the activity on scavenging DPPH radical. Similar to our result, Klompong et al. [52] stated that the yellow strip trevally hydrolysates had better DPPH radical scavenging activity at lower DH. The present results indicated that the SVCE3(f), partly purified cathepsin D-like enzyme from the viscera of neon flying squid, could have a potential application for the production of antioxidant half-fin anchovy hydrolysates.

## 4. Conclusions

A protease from neon flying squid viscera was partly purified by an ISP-UMF process. The optimum pH of ISP was 3.0 to obtain crude protease from neon flying squid viscera. After cutting off < 8 KDa with an ultrafiltration membrane, the protease was finally concentrated 3.98-fold in the collected fraction SVCE3(f), with a protein recovery of 54.55%, and a specific activity of 8.92 U/mg. SDS-PAGE and zymography analysis suggested that at least one protease with molecular weight about 45 KDa existed in SVCE3(f), while UPLC-ESI-MS/MS analysis implied the partial purified protease of SVCE3(f) could be a cathepsin D-like protease. This isolated protease demonstrated an optimum temperature at 60 °C and optimum pH of 3.0 and remained stable at 30–50 °C and pH range of 1.0–5.0. The SVCE3(f) can digest half-fin anchovy to release antioxidant hydrolysates. Furthermore, the DH displayed an important role in the in vitro antioxidant activity of half-fin anchovy hydrolysates. The highest DPPH radical scavenging activity of half-fin anchovy hydrolysates was observed at DH of 8.63% (hydrolysis time of 30 min). By comparison, the greatest hydroxyl radical scavenging activity and reducing power of half-fin anchovy hydrolysates were measured at DH about 15% (hydrolysis time of 60–90 min). Our results suggest that the ISP-UMF process is a convenient method to extract and obtain protease from neon flying squid viscera. The present study also confirmed that the extracted cathepsin D-like protease from fish by-products could be potentially applied in antioxidant hydrolysate production.

## Figures and Tables

**Table 1:** Purification efficiency of enzymes from neon flying squid viscera.

Purification Steps	Protease Activity (U/mL)	Protein Content (mg/mL)	Specific Activity (U/mg)	Purification Fold	Protein Recovery (%)
SVCE	0.49 ± 0.06	0.22 ± 0.04	2.24 ± 0.18	1	100
SVCE3	1.15 ± 0.07	0.14 ± 0.04	8.25 ± 0.74	3.68 ± 0.33	63.64 ± 4.55
SVCE3(f)	1.06 ± 0.09	0.12 ± 0.03	8.92 ± 0.81	3.98 ± 0.36	54.55 ± 9.09

Note: SVCE—squid viscera crude enzymes; SVCE3—SVCEs obtained at pH 3.0; and SVCE3(f)—SVCE3 after ultra-membrane filtration (8 KDa).

**Table 2:** Matched peptide sequences of SVCE3(f) trypsin digest with protein database in NCBI.

Protein Database	Protein Number	Protein	Protein Score	Molecular Weight (Da)	Coverage Ratio	Amino Acid before N-terminus	Sequence	Amino Acid before C-terminus
Teuthida	gi|46309251	Cathepsin D [*Todarodes pacificus*]	1044	42,748	38.5	K	LNEAIGGR	A
						R	KGYWQIK	M
						K	YYTVFDLGK	N
						K	LPNVDFVLGGK	T
						K	EGGELILGGSDPK	H
						K	LSDIACLLHNK	Y
						K	VTPVFYQIISQK	L
						K	LVDQPVFSFYLNR	D
						K	VVFDTGSSNLWVPSK	K
						R	ALPGGEYMVDCASIPK	L
						K	FDGILGMAYDTISVDK	V
						K	VVFDTGSSNLWVPSKK	C
						K	AQTFAEATNQPGLVFVAAK	F
						K	FDGILGMAYDTISVDKVTPVFYQIISQK	L
	gi|315439548	Actin [*Heterololigo bleekeri*]	237	42,111	12.5	R	GYSFTTTAER	E
						K	SYELPDGQVITIGNER	F
						K	DLYANTVLSGGTTMFPGIADR	M
						R	GYSFTTTAER	E
						K	IWHHTFYNELR	V
	gi|223016073	Actin [*T. pacificus*]	73	42,023	9.8	K	SYELPDGQVITIGNER	F
	gi|51860817	Beta actin [*Doryteuthis pealeii*]	73	41,962	9.9	R	GYSFTTTAER	E
						K	IWHHTFYNELR	V
						K	SYELPDGQVITIGNER	F
	gi|1911573	Enolase [*D. pealeii*]	53	47,738	3.5	K	VNQIGSVTESIQACK	M
Coleoidea	gi|46309251	Cathepsin D [*T. pacificus*]	829	42,748	36.7	K	LSDIACLLHNK	Y
						K	VTPVFYQIISQK	L
						K	LVDQPVFSFYLNR	D
						K	VVFDTGSSNLWVPSK	K
						R	ALPGGEYMVDCASIPK	L
						K	FDGILGMAYDTISVDK	V
						K	VVFDTGSSNLWVPSKK	C
						K	AQTFAEATNQPGLVFVAAK	F
						K	FDGILGMAYDTISVDKVTPVFYQIISQK	L
	gi|918295374	Hypothetical protein OCBIM_22000256mg [*Octopus bimaculoides*]	238	42,082	11.2	R	GYSFTTTAER	E
						K	IWHHTFYNELR	V
						K	DLYANTVLSGGTTMYPGIADR	M
	gi|315439550	Actin [*S. esculenta*]	208	41,996	15.4	R	GYSFTTTAER	E
						K	IWHHTFYNELR	V
						K	SYELPDGQVITIGNER	F
						K	DLYANTVLSGGTTMFPGIADR	M
	gi|344944062	Beta-actin [*Sepiella maindroni*]	208	42,238	15.4	R	GYSFTTTAER	E
						K	IWHHTFYNELR	V
						K	SYELPDGQVITIGNER	F
						K	DLYANTVLSGGTTMFPGIADR	M
	gi|385145402	Actin I [*Sepia officinalis*]	208	42,093	15.4	R	GYSFTTTAER	E
						K	IWHHTFYNELR	V
						K	SYELPDGQVITIGNER	F
						K	DLYANTVLSGGTTMFPGIADR	M
	gi|918321201	Hypothetical protein OCBIM_22013417mg [*O. bimaculoides*]	208	42,093	15.4	R	GYSFTTTAER	E
						K	IWHHTFYNELR	V
						K	SYELPDGQVITIGNER	F
						K	DLYANTVLSGGTTMFPGIADR	M
	gi|918314023	Hypothetical protein OCBIM_22023116mg, partial [*O. bimaculoides*]	179	48,773	10.8	K	ESTLHLVLR	L
						K	ESTLHLVLR	L
						R	TLSDYNIQK	E
						K	IQDKEGIPPDQQR	L
						K	TITLEVEPSDTIENVK	A
	gi|918337215	Hypothetical protein OCBIM_22021358mg [*O. bimaculoides*]	67	44,939	2.6	K	NTVGWYECHR	C
	gi|918336596	Hypothetical protein OCBIM_22025091mg, partial [*O. bimaculoides*]	44	45,263	5.7	K	APDSDGGTPLTK	Y
						K	VSYDYVDLEWK	A
						K	VSYDYVDLEWKAPDSDGGTPLTK	Y
	gi|918315574	Hypothetical protein OCBIM_22020904mg [*O. bimaculoides*]	37	43,900	2.1	K	GIIQLLEK	C
	gi|918321870	Hypothetical protein OCBIM_22012740mg [*O. bimaculoides*]	24	43,267	2.7	R	TGATVVDVIR	R
	gi|918297782	Hypothetical protein OCBIM_22038295mg [*O. bimaculoides*]	23	44,186	2.3	K	IGTPVMLLR	N
